# Phage provoke growth delays and SOS response induction despite CRISPR-Cas protection

**DOI:** 10.1098/rstb.2024.0474

**Published:** 2025-09-04

**Authors:** Benoit J. Pons, Urszula Łapińska, Iolanda Lopes-Domingues, Matthew A. W. Chisnall, Edze R. Westra, Stefano Pagliara, Stineke van Houte

**Affiliations:** ^1^ University of Exeter Environment and Sustainability Institute, Penryn TR10 9FE, UK; ^2^ Biosciences, University of Exeter Living Systems Institute, Exeter EX4 4QD, UK

**Keywords:** microbial ecology, phage–bacteria relationships, bacterial defence systems, CRISPR-Cas system, single-cell microscopy, microfluidics

## Abstract

Bacteria evolve resistance against their phage foes with a wide range of resistance strategies whose costs and benefits depend on the level of protection they confer and on the costs for maintainance. *Pseudomonas aeruginosa* can evolve resistance against its phage DMS*3vir* either by surface mutations that prevent phage binding or through CRISPR-Cas immunity. CRISPR immunity carries an inducible cost whose exact origin is still unknown, and previous work suggested it stems from the inability of the CRISPR-Cas system to completely prevent phage DNA injection and subsequent gene expression before clearing the phage infection. However, the bacterial processes involved are still unknown, and we hypothesize that CRISPR-immunity-associated costs could come from increased mortality rate or reduced growth ability compared with surface-resistant bacteria. To tease apart these two mechanisms with divergent ecological consequences, we use a novel microfluidics-based single-cell approach combined with flow cytometry methods to monitor the effects of phage exposure on the survival and growth of its host. We observed that while CRISPR immunity protects from phage-induced lysis, it cannot prevent phage-induced division lag, filamentation and SOS response activation in a subpopulation of the host bacteria. These results suggest that the costs associated with CRISPR immunity at the population level are caused by heterogeneity in phage-induced growth defects.

This article is part of the discussion meeting issue ‘The ecology and evolution of bacterial immune systems’.

## 1. Introduction

One of the major forces driving bacterial evolution is their viral parasites, called bacteriophages (phages) [1]. Indeed, phages are responsible for the lysis of 20–40% of the global bacterial population every day [2] and are estimated to outnumber their hosts by up to 10-to-1 [3], thereby imposing a strong selection pressure on bacterial populations. In response to this pressure, bacteria have evolved a wide array of evasion strategies [4], including anti-phage defence systems [5–7]. Many defence systems only provide innate immunity and are thus rarely involved in the evolution of immunity against new phage threats. Conversely, among the nucleic-acid-targeting defence systems, CRISPR-Cas (clustered regularly interspaced short palindromic repeats, CRISPR-associated) is an adaptive immune system [8–10]. CRISPR-Cas immunity relies on the integration of a fragment of phage genetic material (called a spacer) from a previous failed infection into the bacterial chromosome in a specific CRISPR locus [10]. These loci are then transcribed and processed into CRISPR RNAs (crRNAs) that associate with Cas proteins to form a surveillance complex. Upon a subsequent infection by the same phage, the crRNA is able to recognize sequences matching the spacer, and the surveillance complex can elicit sequence-specific cleavage of the phage genetic material, which prevents phage replication and bacterial lysis. This mechanism thus allows bacteria to quickly acquire resistance against invading phages [11,12]. Alternatively, bacteria can also acquire resistance to phage by altering the surface receptor exploited by phages for adsorption [13,14], a process called surface mutation (SM).

A previous study showed that the type of resistance adopted by the bacterium *Pseudomonas aeruginosa* strain UCPBB-PA14 (PA14) depends on the force of infection exerted by its lytic phage DMS3*vir* [11]. While high phage pressure (i.e. a high phage concentration in the environment) favours resistance through mutation of the receptor in the type IV pilus, lower phage pressure favours CRISPR-Cas immunity through spacer acquisition in the PA14 type I-F CRISPR-Cas system [11]. This observation is at least in part explained by the different fitness costs associated with these resistance strategies. On the one hand, receptor mutations are associated with a constitutive cost, caused by the importance of these receptors in bacterial fitness [13,15,16]. On the other hand, CRISPR-based immunity confers an infection-induced cost [11,17–19] that is not associated with autoimmunity or metabolic costs of *cas* gene expression [18]. Phage DNA injection cannot be blocked by CRISPR immunity, and Meaden *et al.* reported that the PA14 CRISPR-Cas system cannot fully prevent the ensuing phage gene expression before clearing the infection, suggesting this may cause the reduced fitness of the host bacteria [18]. However, the way in which these negative fitness effects manifest within a bacterial population remains unknown.

Here we test two non-mutually exclusive hypotheses to explain the observed inducible costs of CRISPR-based immunity. First, phage pressure could cause an increased mortality rate in CRISPR-immune bacteria compared with SM-resistant bacteria. Second, CRISPR-immune bacteria could suffer from a reduction in their growth rate compared with SM-resistant bacteria. While previous studies did not identify differences in the resistance levels [11] and growth rates [20] associated with these orthogonal resistance strategies, variation in these traits may have been obscured by the relatively low sensitivity of the classical bulk assays that were employed. To overcome these challenges, we analysed the impact of resistance strategies on survival, growth and stress responses in individual bacteria when exposed to phage. Specifically, we employed microfluidics-based single-cell microscopy [21–24] to perform kinetic analysis of individual bacteria across a range of phage exposures. We report that both CRISPR-based and SM-based resistance provide high-level protection against phage in terms of bacterial survival, but at high phage doses, we observe heterogeneity in the phage response of CRISPR-immune bacteria, whereby a subset of CRISPR-immune bacteria undergoes cell division arrest associated with high SOS response activation. These phage-induced growth defects contribute to the inducible costs associated with CRISPR-based immunity.

## 2. Methods

### (a) Bacterial and viral strains

All experiments were based on the strain *P. aeruginosa* UCBPP-PA14 *flgK*::*Tn5B30*(Tc^R^), carrying a single copy of the *Tn5B30*(Tc^R^) transposon stably inserted in the *flgK* gene (referred to as PA14 WT) [25] and its derivatives. This flagellum-defective strain was selected for its impaired motility as it reduces the probability of bacterial escape from the microfluidics channels during the experiment. Bacteria were cultured at 37°C with 180 r.p.m. shaking in LB medium. All cloning steps described in the following sections were performed in *Escherichia coli* DH5α, while *E. coli* S17-1 λ*pir* was used for plasmid conjugation in two-step allelic exchange. Recombinant lytic phages DMS3*vir* and DMS3*mvir*, derived from DMS3*vir* to be targeted by one spacer from the PA14 type I-F CRISPR-Cas system [9], were used throughout this study. Phage stocks were obtained from lysates prepared on a PA14 CRISPR-KO strain [9] and stored at 4°C.

### (b) Generation of PA14 CRISPR knock-out

A functional knock-out of the type I-F CRISPR-Cas system in PA14 was generated by replacing the *csy3* gene of the CRISPR-Cas system with a *lacZ* gene in UCBPP-PA14 *flgK*::*Tn5B30*(Tc^R^) using the previously described two-step allelic exchange protocol [26]. Briefly, 680 bp sequences flanking either side of the PA14 *csy3* gene were synthesized in fusion with the *lacZ* gene (IDT DNA, USA) and cloned into the pDONRpEX18Gm donor vector. This donor vector was electroporated into electrocompetent *E. coli* S17-1 λ*pir*, and the resulting strain was used for conjugation with *P. aeruginosa* UCBPP-PA14 *flgK*::*Tn5B30*(Tc^R^). Merodiploids were selected on cetrimide agar plates with 50 µg ml^−1^ gentamicin and then transferred to LB agar plates supplemented with 15% (w/v) sucrose to select for double crossover clones that had lost the gentamicin marker. The obtained clones were confirmed for *csy3* gene deletion by colony polymerase chain reaction (PCR; using primers csy3_seq_for and csy3_seq_rev, see electronic supplementary material, table S1), followed by Sanger sequencing (Source Biosciences, Cambridge, UK). The confirmed PA14 *flgK*::*Tn5B30*(Tc^R^)*-csy3*::*lacZ* strain is hereafter referred to as PA14 CRISPR-KO.

### (c) Evolution of PA14-derived surface mutant

A surface mutant derived from PA14 *flgK*::*Tn5B30*(Tc^R^) was evolved through infection with DMS3*vir* as previously described [11]. An overnight culture of PA14 *flgK*::*Tn5B30*(Tc^R^*)* grown in LB medium was diluted 100-fold in fresh LB medium to approximately 10^7^ colony-forming units (cfu) ml^−1^ and simultaneously infected with 10^6^ plaque-forming units (pfu) ml^−1^ of DMS3*vir*. Cultures were diluted 100-fold in fresh LB medium every day for 3 days, after which individual clones were isolated and streaked through DMS3*vir* solution to check their phage resistance phenotype. Surface mutation was confirmed through a resistant phenotype against DMS3*vir* and by colony morphology (loss of twitching motility). Colony PCR (using primers CRISPR1_for and CRISPR1_rev or CRISPR2_for and CRISPR2_rev, see electronic supplementary material, table S1) was performed on the positive clones to confirm that no spacer was acquired, so as to exclude that the observed resistance phenotype was due to CRISPR-Cas immunity. Whole-genome sequencing was performed on one mutant displaying the correct resistance phenotype to identify the underlying mutations, and the resulting sequence (electronic supplementary material, data S2) was analysed with Snippy v. 4.6.0 (https://github.com/tseemann/snippy) against the PA14 reference genome (NCBI: CP000438.1) to identify mutations in the evolved clone. The list of mutations was matched with PA14 genome annotations to highlight the name and function of mutated coding sequences (electronic supplementary material, data S3). Among the listed mutations, we identified a loss-of-function insertion in the *pilP* gene (electronic supplementary material, figure S1), a gene involved in the formation of the PA14 type IV pilus [27], the bacterial surface structure to which DMS3*vir* attaches [28].

The confirmed surface-mutated strain derived from PA14 *flgK*::*Tn5B30*(Tc^R^) is hereafter referred to as PA14 SM. Finally, the absence of phage attachment to the cell surface of the surface mutant was verified through an adsorption assay (electronic supplementary material, figure S1), with a protocol adapted from [29]. Briefly, 10 ml of PA14 *flgK*::*Tn5B30*(Tc^R^) WT or surface mutant culture (OD_600_ = 0.1, approx. 1 × 10^8^ cfu ml^−1^) was infected with 10^5^ pfu ml^−1^ of phage DMS3*mvir*. Infection was carried out at 37°C with 180 r.p.m. shaking, and a control without bacteria was assessed in parallel. A 200 µl aliquot was sampled every 5 min for 35 min and then transferred to a chilled microtube containing 100 µl of chloroform. Tubes were vortexed upon sample transfer and centrifuged for 1 min at 15 000*g* to ensure a proper separation between free phages and bacteria. Phage-containing supernatants were serially diluted and spotted on PA14 CRISPR-KO lawns to determine phage titre over time, which confirmed that DMS3*mvir* did not adsorb to PA14 *flgK*::*Tn5B30*(Tc^R^) surface mutant.

### (d) Generation of PA14 SOS response reporter strains

To measure SOS response induction, the *lexA* promoter was PCR-amplified (using primers pLex_for and pLex_rev; see electronic supplementary material, table S1) from *P. aeruginosa* UCBPP-PA14 and subsequently cloned upstream of the *gfp* gene in the pHERD30T vector to construct p*lexA-gfp*. The obtained plasmid was then electroporated into the three different PA14 *flgK*::*Tn5B30*(Tc^R^) strains (PA14 *flgK*::*Tn5B30*(Tc^R^), PA14 *flgK*::*Tn5B30*(Tc^R^) surface mutant and PA14 *flgK*::*Tn5B30*(Tc^R^) *csy3*::*lacZ*), which were previously made electrocompetent through 300 mM sucrose washes [30].

### (e) Phage infection assays in a microfluidic environment

The mould for the mother machine microfluidic device was fabricated by Kelvin Nanotechnology using previously established multi-level photolithography processes [31]. This mould is equipped with six identical microfluidic networks that can be controlled simultaneously and independently to maximize experimental throughput [32]. Each of these networks is equipped with approximately 6000 lateral microfluidic channels with a width and height of 1 μm each and a length of 20 μm. These lateral channels are connected to a main microfluidic chamber that is 25 and 100 μm in height and width, respectively. Polydimethylsiloxane (PDMS) replicas of this device were realized as previously described [33]. Briefly, a 10 : 1 (base:curing agent) PDMS mixture was cast on the mould and cured at 70°C for 120 min in an oven. The cured PDMS was peeled from the epoxy mould, and fluidic accesses were created by using a 0.75 mm biopsy punch (Harris Uni-Core, WPI). The PDMS chip was irreversibly sealed on a glass coverslip by exposing both surfaces to oxygen plasma treatment (10 s exposure to 30 W plasma power plasma etcher, Diener, Royal Oak, MI, USA; [34]). This treatment temporarily rendered the PDMS and glass hydrophilic, so immediately after bonding, the chip was filled with bacteria. We have also made available a step-by-step experimental protocol for the fabrication and handling of microfluidic devices for investigating the interactions between antibiotics and individual bacteria [35].

Overnight cultures of the PA14 flgK::Tn5B30(Tc^R^)*,* PA14 flgK::Tn5B30(Tc^R^)-csy3::lacZ or PA14 flgK::Tn5B30(Tc^R^) surface mutant were centrifuged at 4000 r.p.m. (4800 g) for 5 min and resuspended in twice-filtered supernatant to an OD_600_ = 70. A 2 µl sample of these concentrated bacteria was injected into the mother machine device and incubated at 37°C until each growth channel contained 3 or 4 bacteria. Fluorinated ethylene propylene tubing (1/32″ x 0.008″) was connected to the inlet and outlet holes of the main microfluidic channel and connected to a computerized pressure-based flow control system (MFCS-4C, Fluigent) controlled by MAESFLO software (Fluigent) and an outlet reservoir, respectively [36]. When each channel contained 3 or 4 bacteria, fresh LB was flushed through the device at 300 µl h^−1^ for 5 min to wash excess bacteria out of the main channel. The mother machine device was then mounted on an inverted microscope (IX73 Olympus, Tokyo, Japan), and images were acquired in bright field via a 60×, 1.2 N.A. objective (UPLSAPO60XW, Olympus) and an sCMOS camera (Zyla 4.2, Andor, Belfast, UK) with a 0.03 s exposure [37]. The microfluidic device was moved by two automated stages (M-545.USC and P-545.3C7, Physik Instrumente, Karlsruhe, Germany, for coarse and fine movements, respectively) to image multiple fields of view in a sequential manner. The imaging setup was controlled by LabView. After setting up the imaging, LB or LB containing 10^7^ or 10^10^ pfu ml^−1^ of phages DMS3*vir* or DMS3*mvir* was supplied to the main microfluidic channel at 100 µl h^−1^ for 7 h with image acquisition approximately every 5 min. The whole experiment was carried out at 37°C in an environmental chamber surrounding the microscope [38].

### (f) Single-cell microscopy image analysis

Time-lapse microscopy images acquired in the microfluidics experiments were quantitatively analysed using ImageJ software as previously described [39]. Briefly, individual bacteria were manually tracked throughout the experiment to assess their fate: division (considered to have happened when two daughter cells became clearly distinguishable from the parental cell), departure from the microfluidics channel (considered to have happened when bacteria disappeared while being outermost in the microfluidic channel) or lysis (considered to have happened when visible cell debris was observable where the bacterium was previously present, or when bacteria disappeared from the channel while not being outermost in the microfluidic channel). In addition, the bacterial length was determined to assess the filamentation status, with a threshold of 6 µm to differentiate between filamentous and non-filamentous bacteria. For each experiment, data were analysed until at least 50 bacteria of known fate (lysis or survival) were assessed.

### (g) SOS response assays through flow cytometry

Overnight cultures of the three strains carrying p*lexA-gfp* grown in LB medium supplemented with 50 µg ml^−1^ gentamicin were diluted 100-fold in fresh medium to approximately 10^7^ cfu ml^−1^ and either left untreated or treated with 1 µg ml^−1^ mitomycin C (MMC) or 10^7^ pfu ml^−1^ phage. After 10 h of culture, cells were harvested by centrifugation and washed twice in phosphate-buffered saline (PBS). Cells were diluted 100× in PBS and analysed using an automated sample loader on a five-laser Cytek Aurora full-spectrum flow cytometer (Cytek, USA). Cytometry data were analysed with FlowJo software (BD, USA). Bacteria were gated to separate cells from debris and single bacteria from aggregates with the forward scatter (FSC) and side scatter (SSC) signals. A GFP–FSC plot of single bacteria was then graphed, and three subpopulations were gated: ‘regular-sized’ bacteria that are not elongated and have low GFP signal, ‘filamentous GFP−’ with a high FSC signal but a low GFP signal and ‘filamentous GFP+’ with both high FSC and GFP signal.

### (h) Quantification and statistical analysis

For microfluidics experiments, single replicates of each strain–phage treatment combination were performed, except the PA14 WT conditions, for which two independent replicates were performed (table 1) in order to verify data replicability (electronic supplementary material, figure S6). Three independent biological replicates of the cytometry experiments were performed. Growth rates of individual bacteria were calculated with the following formula (with *R*, growth rate in h^−1^, and *t*, division time in h):


$$R\;=\frac{ln2}{t}.$$

**Table 1.** Bacteria–phage combinations were tested in the mother machine. Each ‘X’ indicates a technical replicate.

	PA14 WT	PA14 SM	PA14 CRISPR-KO
no phage	XX	X	X
DMS3*mvir* 10^7^ pfu ml^−1^	XX	X	X
DMS3*mvir* 10^10^ pfu ml^−1^	XX	X	X
DMS3*vir* 10^7^ pfu ml^−1^	XX		
DMS3*vir* 10^10^ pfu ml^−1^	XX		

**Figure 1.**
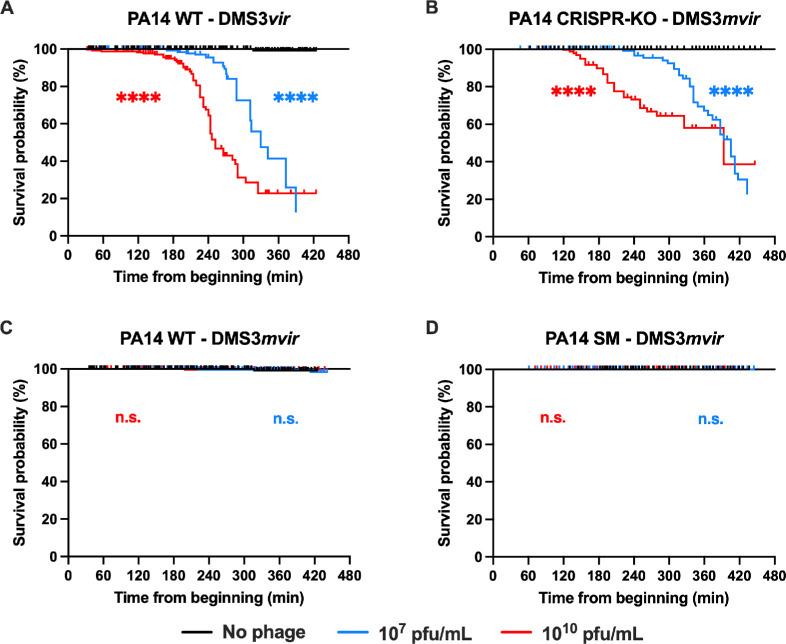
CRISPR-based immunity is as effective as surface mutation (SM)-based resistance. Survival analysis of individual bacteria as observed in microfluidics in the absence (black line) or presence of 10^7^ pfu ml^−1^ (blue line) or 10^10^ pfu ml^−1^ (red line) of the indicated phage. Visible bacterial lysis was considered an occurred event, while bacteria leaving the microfluidics channel was considered a censored event. Time is counted from the beginning of the experiment. (A) PA14 WT (CRISPR-immune) strain exposed to phage DMS3*vir* (non-targeted; no phage, *n* = 345; 10^7^ pfu ml^−1^, *n* = 473; 10^10^ pfu ml^−1^, *n* = 270). (B) PA14 CRISPR-KO strain exposed to phage DMS3*mvir* (CRISPR-targeted; no phage, *n* = 629; 10^7^ pfu ml^−1^, *n* = 454; 10^10^ pfu ml^−1^, *n* = 313). (C) PA14 WT (CRISPR-immune) strain exposed to phage DMS3*mvir* (CRISPR-targeted; no phage, *n* = 345; 10^7^ pfu ml^−1^, *n* = 326; 10^10^ pfu ml^−1^, *n* = 274), and (D) PA14 SM strain exposed to phage DMS3*mvir* (CRISPR-targeted; no phage, *n* = 252; 10^7^ pfu ml^−1^, *n* = 361; 10^10^ pfu ml^−1^, *n* = 276). Asterisks show conditions that are different from the ‘no phage’ treatment (Bonferroni-corrected Mantel–Cox *p*‐value: n.s., *p* > 0.05; ****0.0001 > *p*).

**Figure 2.**
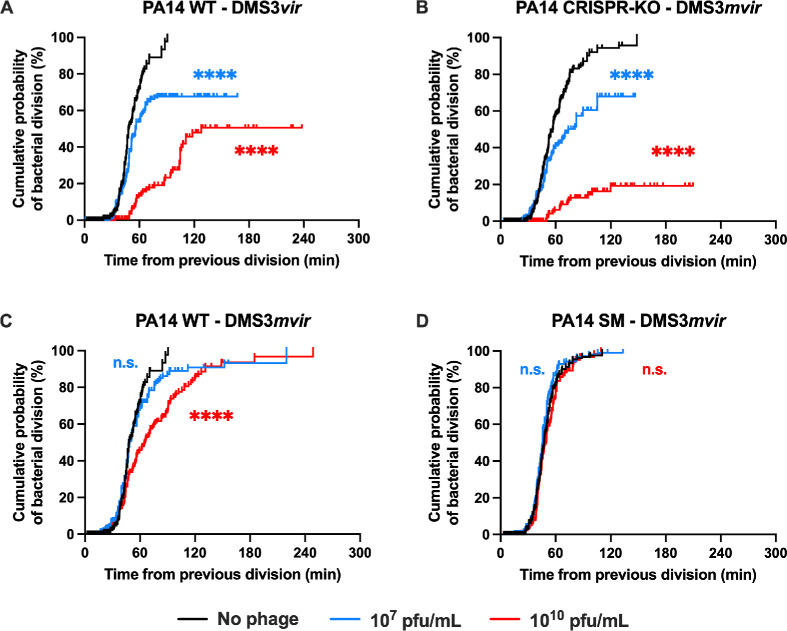
Phage treatment induces division lag in sensitive and CRISPR-immune bacteria but not in surface mutation (SM)-resistant bacteria. Kaplan–Meier analysis of individual bacteria division as observed in microfluidics in the absence (black line) or presence of 10^7^ pfu ml^−1^ (blue line) or 10^10^ pfu ml^−1^ (red line) of the indicated phage. Visible bacterial division was considered an occurred event while bacteria dying or leaving the microfluidics channel was considered a censored event. Time is counted from the previous division. (A) PA14 WT (CRISPR-immune) strain exposed to phage DMS3*vir* (non-targeted; no phage, *n* = 355; 10^7^ pfu ml^−1^, *n* = 588; 10^10^ pfu ml^−1^, *n* = 200). (B) PA14 CRISPR-KO strain exposed to phage DMS3*mvir* (CRISPR-targeted; no phage, *n* = 730; 10^7^ pfu ml^−1^, *n* = 450; 10^10^ pfu ml^−1^, *n* = 148). (C) PA14 WT (CRISPR-immune) strain exposed to phage DMS3*mvir* (CRISPR-targeted; no phage, *n* = 355; 10^7^ pfu ml^−1^, *n* = 445; 10^10^ pfu ml^−1^, *n* = 329), and (D) PA14 SM strain exposed to phage DMS3*mvir* (CRISPR-targeted; no phage, *n* = 407; 10^7^ pfu ml^−1^, *n* = 582; 10^10^ pfu ml^−1^, *n* = 416). Asterisks show conditions that are different from the ‘no phage’ treatment (Bonferroni-corrected Mantel–Cox *p*‐value: n.s., *p* > 0.05; ****0.0001 > *p*).

**Figure 3.**
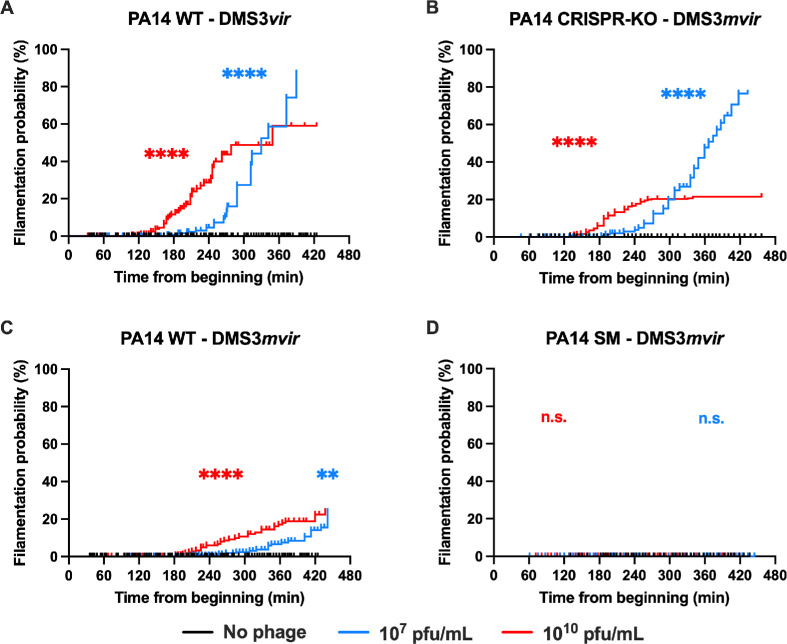
Phage treatment provokes bacterial filamentation in sensitive and CRISP-immune bacteria but not in surface mutation (SM)-resistant bacteria. Kaplan–Meier analysis of individual bacteria filamentation as observed in microfluidics in the absence (black line) or presence of 10^7^ pfu ml^−1^ (blue line) or 10^10^ pfu ml^−1^ (red line) of the indicated phage. Bacteria exceeding 6 µm were considered filamentous and counted as an occurred event, while bacteria dying or leaving the microfluidics channel were considered a censored event. Time is counted from the beginning of the experiment. (A) PA14 WT (CRISPR-immune) strain exposed to phage DMS3*vir* (non-targeted; no phage, *n* = 345; 10^7^ pfu ml^−1^, *n* = 473; 10^10^ pfu ml^−1^, *n* = 270). (B) PA14 CRISPR-KO strain exposed to phage DMS3*mvir* (CRISPR-targeted; no phage, *n* = 629; 10^7^ pfu ml^−1^, *n* = 454; 10^10^ pfu ml^−1^, *n* = 313). (C) PA14 WT (CRISPR-immune) strain exposed to phage DMS3*mvir* (CRISPR-targeted; no phage, *n* = 345; 10^7^ pfu ml^−1^, *n* = 326; 10^10^ pfu ml^−1^, *n* = 274), and (D) PA14 SM strain exposed to phage DMS3*mvir* (CRISPR-targeted; no phage, *n* = 252; 10^7^ pfu ml^−1^, *n* = 361; 10^10^ pfu ml^−1^, *n* = 276). Asterisks show conditions that are different from the ‘no phage’ treatment (Bonferroni-corrected Mantel–Cox *p*‐value: n.s., *p* > 0.05; **0.01 > *p* > 0.001; ****0.0001 > *p*).

Kaplan–Meier analysis of bacterial survival (figure 1) was performed, with bacterial lysis being counted as an event and bacteria departure from the channel counted as a censored event, while Kaplan–Meier analysis of bacterial division probability (figure 2) was performed by considering bacterial division as an event and bacteria lysing or leaving the channel as a censored event. Similarly, Kaplan–Meier analysis of bacterial filamentation probability (figure 3) was performed by considering bacterial filamentation as an event and bacteria lysing or leaving the channel as a censored event.

**Figure 4.**
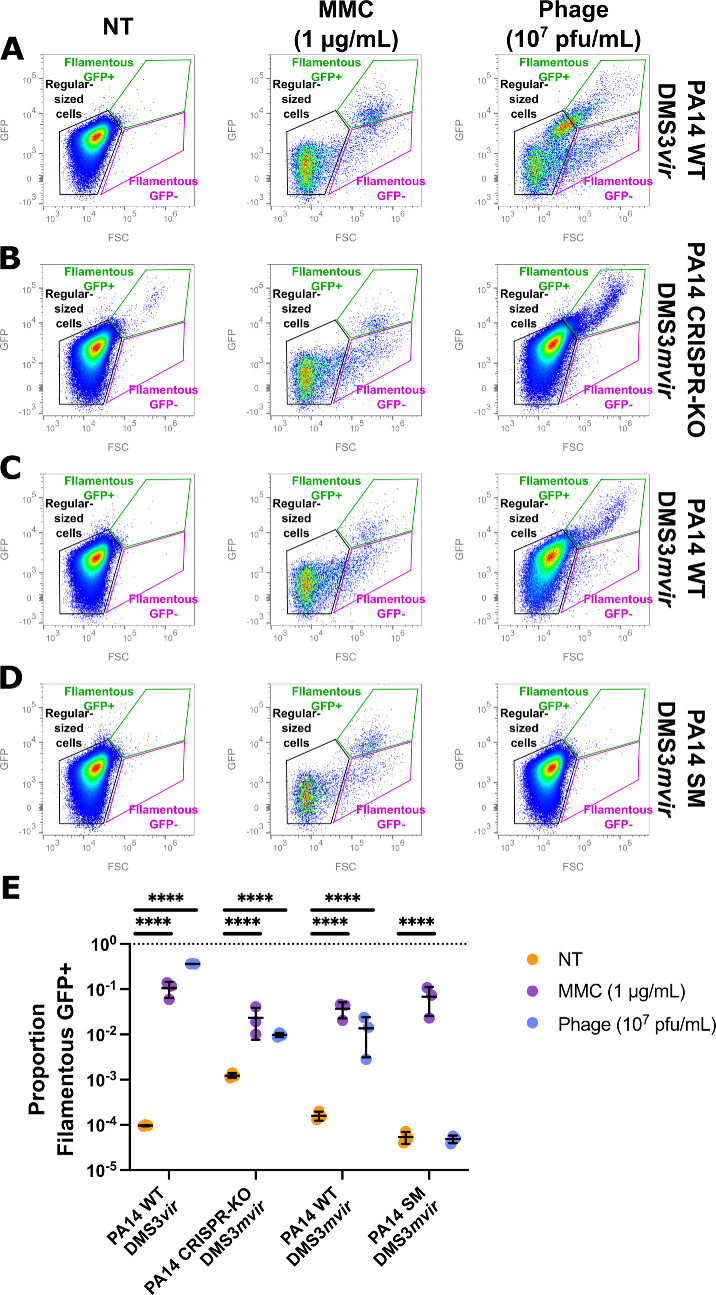
Phage treatment induces the SOS response in sensitive and CRISPR-immune bacteria but not in surface mutation (SM)-resistant bacteria. Flow cytometry analysis of individual bacteria carrying a plexA-GFP reporter plasmid. (A–D) Pseudocolour bivariate density plot of forward light scattering (FSC) signal versus GFP signal of non-treated bacteria (NT) and of bacteria exposed to 1 µg ml^−1^ mitomycin C (MMC) or to 10^7^ pfu ml^−1^ of the different phage treatments. Three subpopulations were determined: regular-sized cells (black), GFP-negative elongated cells (pink) and GFP-positive elongated cells (green). Representative images of a single technical replicate (*n* = 3). (A) PA14 WT (CRISPR-immune) strain exposed to phage DMS3*vir* (non-targeted), (B) PA14 CRISPR-KO strain exposed to phage DMS3*mvir* (CRISPR-targeted), (C) PA14 WT (CRISPR-immune) strain exposed to phage DMS3*mvir* (CRISPR-targeted), (D) PA14 surface mutant strain exposed to phage DMS3*mvir* (CRISPR-targeted), and (E) proportion of GFP-positive elongated bacteria (as determined in (A–D)) either left untreated (NT, orange) or exposed to 1 µg ml^−1^ MMC (purple) or to 10^7^ pfu ml^−1^ of the different phage treatments (phage, blue*; n* = 3). Asterisks show conditions that are different from the NT condition (Dunnett two-way ANOVA *p*‐value: n.s., *p* > 0.05; **** 0.0001 > *p*).

All statistical analyses were performed in GraphPad Prism v. 10.4.0, and statistical parameters are reported in the figure legends or in the Results. For Kaplan–Meier analyses of individual bacterial survival, division and filamentation, pairwise comparisons between each phage treatment and the corresponding ‘no phage’ control were made in order to obtain individual log-rank (Mantel–Cox) *p*-values, which were then adjusted using Bonferroni correction to account for multiple comparisons. Statistical differences in growth rate were assessed through one-way ANOVA with Tukey *post hoc* test (electronic supplementary material, figure S2), while two-way ANOVA with Dunnett *post hoc* test was performed on the log-transformed proportion of cells (figure 4E).

## 3. Results

### (a) CRISPR immunity is not associated with an increased mortality rate

To study phage–bacteria interactions at a single-cell level, we used a mother machine device to perform microfluidic-based microscopy that was previously used to study phage–bacteria relationships [21,22,40,41]. Since *P. aeruginosa* PA14 is a highly motile bacterium [25] that is able to swim out of the spatial refuges, we used PA14 *flgK*::*Tn5B30*(Tc^R^) to prevent swimming motility via the flagellum. We compared three different derivative strains (table 1): PA14 *flgK*::*Tn5B30*(Tc^R^) WT (with a functional CRISPR-Cas system), PA14 *flgK*::*Tn5B30*(Tc^R^) SM (resistant to phage infection through surface mutation) and PA14 *flgK*::*Tn5B30*(Tc^R^)-*csy3*::*lacZ* (without CRISPR immunity nor SM-resistance), henceforth PA14 WT, PA14 SM and PA14 CRISPR-KO, respectively. These strains were subjected to different phage treatments within LB medium continuously supplied to the bacteria (table 1): no phage, 10^7^ pfu ml^−1^ (low dose) or 10^10^ pfu ml^−1^ (high dose) of phages DMS3*vir* (blocked by SM-based resistance but not targeted by PA14 WT CRISPR-Cas system) or DMS3*mvir* (blocked by SM-based resistance and targeted by one spacer from PA14 WT CRISPR-Cas system). These experiments allowed us to determine the kinetics of bacterial growth and mortality at the single-cell level and to specifically tease out the effect of phage exposure to cells that are immune to the phage via CRISPR-Cas.

In order to test our first hypothesis (CRISPR-immune bacteria exhibit a higher phage-induced mortality than SM-resistant bacteria), we determined the time needed from the beginning of the experiment for each bacterium to either lyse (see Methods) or leave the channel (figure 1). When PA14 WT and PA14 CRISPR-KO were exposed to phage to which they were sensitive (DMS3*vir* and DMS3*mvir*, respectively), bacterial survival probability decreased after 3–4 h, depending on the phage dose (figure 1A,B). Conversely, both PA14 WT and PA14 SM were able to fully survive both high and low doses of phage DMS3*mvir* (figure 1C,D), thus showing that both resistance strategies (CRISPR-Cas immunity versus SM-resistance) fully protect against DM3*mvir* in this setting. Therefore, this result does not support the hypothesis that the induced fitness cost of CRISPR immunity is caused by an increased mortality rate in CRISPR-immune bacteria compared with SM-resistant bacteria during phage exposure.

### (b) CRISPR immunity is associated with phage-mediated growth defects

To test the hypothesis that CRISPR-immune bacteria have a reduced growth rate compared with SM-resistant bacteria in the presence of phage, we determined the time between two consecutive bacterial cell divisions and used it to calculate individual bacteria growth rates (cells that enter into growth arrest are thus excluded from the analysis, or ‘censored’, see Methods; electronic supplementary material, figure S2). As expected, we found that high doses of phages cause a reduced growth rate in sensitive bacteria. Interestingly, no statistically significant effect on the growth rate of resistant bacteria was observed, irrespective of the resistance strategy of the bacteria (CRISPR or SM; electronic supplementary material, figure S2). However, we noticed that a subpopulation of CRISPR-immune bacteria stopped dividing for an extended amount of time before either dying or leaving the channel.

To quantify the contribution of these cells to the overall population growth, we re-analysed the data by measuring for each bacterium the time needed to divide, leave the channel or lyse, from the beginning of the experiment (for the first observed division, i.e. the lag time) or from the previous division (for all divisions following the first one). We performed a Kaplan–Meier analysis on these data, and determined a cumulative probability of bacteria experiencing a first division (electronic supplementary material, figure S3) and a cumulative probability of bacterial dividing for all other consecutive divisions (figure 2). When sensitive bacteria were infected with phages they were not immune to (i.e. PA14 WT infected with DMS3*vir*; PA14 CRISPR-KO infected with DMS3*mvir*; figure 2A,B, respectively), we observed that the cumulative probability of bacterial division did not reach 100% (table 2), indicating that a significant proportion of bacteria stopped dividing after their first division. Conversely, when bacteria were immune to the phage treatment, the cumulative probability of division reached 100% in both cases (PA14 WT and PA14 SM infected with DMS3*mvir*; figure 2C,D, respectively).

**Table 2.** Kaplan–Meier analysis parameters for bacterial division probability as calculated in figure 2.

Bacterial strain	Phage treatment	median division time (min)	maximum division probability (%)
PA14 WT	no phage	48.4	100
PA14 WT	DMS3*vir* 10^7^ pfu.mL^-1^	51.4	67.6
PA14 WT	DMS3*vir* 10^10^ pfu.mL^-1^	104.5	50.6
PA14 WT	DMS3*mvir* 10^7^ pfu.mL^-1^	48.6	100
PA14 WT	DMS3*mvir* 10^10^ pfu.mL^-1^	65.8	100
PA14 SM	no phage	47.4	100
PA14 SM	DMS3*mvir* 10^7^ pfu.mL^-1^	45.7	100
PA14 SM	DMS3*mvir* 10^10^ pfu.mL^-1^	49.7	100
PA14 CRISPR-KO	no phage	57.0	100
PA14 CRISPR-KO	DMS3*mvir* 10^7^ pfu.mL^-1^	69.5	67.9
PA14 CRISPR-KO	DMS3*mvir* 10^10^ pfu.mL^-1^	119.9	19.2

In addition, we determined a median division time at which the division probability is equal to one-half of the maximum division probability (table 2). We observed that phage treatment increased the median division time of sensitive bacteria (figure 2A,B), showing that they suffer from growth defects when they are sensitive to the phage treatment (table 2). Interestingly, PA14 WT showed a similar behaviour when in contact with the higher dose of DMS3*mvir* phage, even though it is immune to this phage (figure 2C and table 2), but SM-resistant bacteria were protected against these growth defects even at high phage dose (figure 2D and table 2). Therefore, SM-based resistance offers full protection from bacterial growth defects caused by phage infection, while CRISPR-based immunity does not. Similar results were observed for the probability of reaching the first division (electronic supplementary material, figure S3).

These data therefore confirm the hypothesis that the induced fitness cost associated with CRISPR-Cas immunity is due to a reduced growth rate of the population, which is caused by growth arrest of a subpopulation that exists alongside cells that continue to replicate at a rate that is unaffected by the presence of the phage.

### (c) Phage infection induces SOS response despite CRISPR immunity

We also noted that in some of the tested conditions, a subpopulation of bacteria became filamentous (electronic supplementary material, figure S4A), particularly among bacteria experiencing division lag (electronic supplementary material, figure S5). Since regular-size bacteria just before division are 4.2 µm long on average and are never longer than 5.8 µm, we defined a 6 µm threshold to differentiate between filamentous and non-filamentous bacteria. We quantified the filamentation of bacteria and performed a Kaplan–Meier analysis on these data (figure 3). Filamentation was not observed in any strain (WT, CRISPR-KO, SM) in the absence of phage, nor in the SM-resistant strain during phage exposure. However, filamentation was observed in PA14 WT subjected to either DMS3*vir* or DMS3*mvir*, as well as in PA14 CRISPR-KO exposed to DMS3*mvir*. This shows that filamentation is triggered by phage exposure in both sensitive and CRISPR-immune bacteria.

Filamentation is often linked with bacterial stress response [42–45] and, in particular, the SOS response [46], a global response to DNA damage involving cell cycle arrest and activation of DNA repair mechanisms [47]. We thus hypothesize that the observed filamentation is caused by a bacterial stress response such as the SOS response. Based on a previously published reporter system [48], we built a SOS reporter plasmid carrying an *egfp* gene under the control of the promoter region of the PA14 *lexA* gene, a gene responsible for SOS-response self-regulation [47]. This reporter plasmid was introduced in PA14 WT, PA14 SM and PA14 CRISPR-KO, which were grown without treatment, in the presence of MMC, a known potent SOS response inducer [47] or in the presence of 10^7^ pfu ml^−1^ phage (either DMS3*vir* or DMS3*mvir*). These bacteria were then analysed using flow cytometry to quantify GFP fluorescence of individual bacteria as well as their FSC signal, which can be used as a proxy for cell size [49]. This revealed that control (i.e. non-treated) bacteria are a single homogeneous population, whereas bacteria treated with MMC displayed three subpopulations: ‘regular-sized’ bacteria that are not elongated and have low GFP fluorescence signal, ‘filamentous GFP’ with a high FSC signal but a low GFP fluorescence signal, and ‘filamentous GFP+’ with both high FSC and GFP signal (figure 4). When subjected to phage, only PA14 WT and PA14 CRISPR-KO displayed the three subpopulations similar to those seen in the MMC treatment, while PA14 SM was not affected by the presence of the phage (figure 4). The increase in the proportion of ‘elongated GFP+’ bacteria (figure 4E) suggests that the SOS response plays an important role in the filamentation phenotype observed upon phage treatment. However, an increase in the ‘filamentous GFP−’ population (electronic supplementary material, figure S4B) implies that the SOS response is not the only responsible mechanism. Overall, these data support the hypothesis that the activation of bacterial stress responses upon phage infection causes growth delays in CRISPR-immune bacteria but not in SM-resistant bacteria.

## 4. Discussion

Previous work showed that while PA14 evolves immunity against low doses of DMS3*vir* by acquiring new CRISPR-Cas spacers, higher phage doses strongly favour SM-based resistance [11]. This led to the conclusion that, unlike the SM-associated fixed fitness cost [13,15,16], CRISPR immunity comes with an inducible cost [11,17]. In order to understand the origin of CRISPR-associated inducible costs, we sought to determine the costs and benefits associated with both resistance mechanisms using single-cell microscopy. However, we noticed that the proportion of bacteria leaving the channels seemed to vary between experiments. We thus sought to assess the robustness and replicability of this method by comparing two technical replicates for each condition relying on the PA14 WT strain (electronic supplementary material, figure S6 and table S2). The most notable differences between replicates can be seen for the maximum probability of division (electronic supplementary material, table S3), which can be explained by variations in the number of cells leaving the channels, but the Kaplan–Meier curves constructed for all parameters (survival, division and filamentation) are qualitatively very similar.

We formulated and assessed two different hypotheses regarding the cause of the phage-induced cost of CRISPR immunity. First, we tested if CRISPR-immune bacteria suffered from an increased phage-induced mortality compared with SM-resistant bacteria. We did not observe any difference in bacterial survival between the two resistance strategies (figure 1), which does not support this first hypothesis. We observed that in the absence of CRISPR immunity (figure 1A,B), the survival rate by the end of the experiment appears higher for the higher phage dose than for the lower one. This phenomenon reflects the fact that some bacteria do not lyse or leave the channel until the end of the experiment when submitted to a high phage dose but not with a lower phage dose. While these bacteria did not display lysis, we cannot exclude that they are dead despite retaining a regular shape, as previously observed [21].

Second, we hypothesized that phage treatment could provoke greater growth defects in CRISPR-immune bacteria compared with SM-resistant bacteria. We tested this second hypothesis by comparing the bacterial median time to division in both strains. We showed that while SM-resistant bacteria are unaffected by phage treatment, CRISPR-immune bacteria suffer from significant growth delays at higher phage doses (figure 2). We also observed heterogeneity in the CRISPR-immune population, with a significant proportion of cells suffering from division lag and filamentation (figure 3). Finally, we showed that this filamentation is at least in part linked with activation of the SOS response upon phage infection (figure 4).

Overall, we observed that phage infection causes SOS response activation, filamentation and growth defects even if it is cleared by the CRISPR-Cas system, but preventing the phage from binding by SM-resistance allows bacteria to avoid all these adverse effects. This suggests that phage DNA injection and subsequent phage gene expression, which cannot be fully prevented by the CRISPR-Cas system, as previously shown by RNAseq [18], could cause SOS response activation, which is known to block bacterial division and thereby cause filamentation [46]. Interestingly, the response to phage is heterogeneous, with a majority of unaffected bacteria that do not suffer from phage presence and a subpopulation undergoing filamentation and division lag. A similar morphological and division heterogeneity was observed in *E. coli* in response to ciprofloxacin, an SOS-inducing antibiotic [45]. The phage-induced heterogeneity explains why the measured growth defects do not prevent CRISPR immunity from evolving at low phage dose (when SOS response activation and growth defects are scarce), but switch the balance in favour of SM-resistance at higher phage dose (when SOS response activation and growth defects are more common).

Both biotic and abiotic complexity were shown to impact phage–host interactions and coevolution [1], and the experimental setting used here is necessarily simpler than a natural environment or clinical setting. Indeed, we used here a single phage–bacterium pair, while in natural environments, bacteria and phage are typically part of a complex microbial community. For instance, in biofilms, bacteria can coexist with phage they are sensitive to [50] and with other bacterial species [51]. Therefore, we can only speculate that the phage-induced SOS response would be different in such a complex environment. Similarly, the phage-induced SOS response could affect interactions of the host with other phages or prophages. Indeed, the SOS response is a known activator of prophages [47], and it can therefore be speculated that phage infection could influence the lysis/lysogeny balance of competitor prophages [52], akin to mammalian virus reactivation upon superinfection [53]. Moreover, filamentation can sensitize the host cell to phage predation by increasing the cell surface [54]. Therefore, even if the bacterial host survives phage infection through CRISPR immunity, phage-induced filamentation could sensitize it to predation by other phages. Overall, these phenomena suggest that phage-induced SOS response and filamentation can influence the relationship of the bacterial host with other phage parasites.

Even if the CRISPR-Cas system prevents bacterial lysis, a phage-induced SOS response could have important consequences for the host bacteria. Indeed, SOS response activation can rewire the bacterial gene expression networks [47], potentially leading to mutagenesis [55], which suggests yet another role of phage in shaping the genome and evolution of their bacterial hosts. Moreover, given that the SOS response can facilitate the acquisition of antibiotic resistance [56], phage-induced SOS response could hamper the efficiency of phage–antibiotic combined therapy.

SOS response induction upon phage infection was previously observed in *Salmonella enterica* with phages P22 and SE1 [57], in *Rhodococcus erythropolis* with phage WC1 [58] and in *E. coli* with phages ΦX174 [59] and lambda [52]. However, the exact mechanisms behind phage-induced SOS response are still unknown. SOS response activation could be promoted by a specific phage gene product. For instance, the *kil* phage gene is responsible for *E. coli* filamentation provoked by phages lambda [49,60,61], Mu [62] and Rac [63], as well as SOS response activation in *S. enterica* by phages P22 and SE1 [57]. The product of this gene was shown to interfere with the normal functioning of the FtsZ protein ring [61,63], a structure at the nascent division site that is essential for bacterial division [64,65]. Additionally, the SOS response could be induced by the bacterial immune response itself [66–68]. Indeed, the normal function of DNA-targeting defence systems such as CRISPR-Cas or restriction–modification leads to the generation of DNA fragments, and these fragments could potentially be mistakenly recognized as damaged host DNA by the SOS response. Consistent with this hypothesis, we observed a higher probability of bacterial filamentation (figure 3) and a stronger SOS response induction (figure 4) in CRISPR-immune PA14 WT exposed to DMS3*vir* non-targeted phage than in the PA14 CRISPR-KO strain exposed to DMS3*mvir* phage.

Interestingly, our flow cytometry experiments reveal the existence of a filamentous subpopulation that does not exhibit SOS response activation (electronic supplementary material, figure S4), thus suggesting that the SOS response is not the only player responsible for bacterial filamentation and division lag. Such SOS-independent filamentation was previously observed in response to the expression of gene *P* from phage lambda [69], the *dicB* gene from *E. coli* defective prophage Qin [70–72], and *gp46* from phage SPO1 in *Bacillus subtilis* [73,74]. The proteins encoded by these genes provoke cell division arrest by interacting with diverse proteins involved in DNA replication [69], cytokinesis [71] or nucleoid formation [74]. This is consistent with the normal progress of phage infection, where most of the normal host processes are shut off to prevent competition with the phage replication. Therefore, we cannot exclude that a bacterial subpopulation undergoes division lag and filamentation upon phage gene expression, not because of SOS response activation but because of the intrinsic activity of these gene products on bacterial division processes.

In order to face threats such as viral infections, organisms have evolved a wide variety of defence systems. They can be divided into constitutive defences that are always active and inducible defences that are only elicited upon infection [75]. Fitness costs associated with each type of defence are often linked with activity: constitutive defences carry a fixed cost [76,77] while the cost of inducible defences is linked with the infection risk [78]. Theory predicts that these inducible costs can be different in nature: caused by either a reduced growth rate, a reduced reproductive output or an impaired survivorship [79]. Since the reproductive output of bacteria is fixed, we assume here that the balance between constitutive and inducible defences might be only influenced by the growth rate and survival of bacteria when adopting one or the other strategy. We showed here that the balance between CRISPR immunity and SM-resistance is mediated by the growth rate defects imposed by the phage infection, and thus confirm the model prediction of an ‘immune lag cost’ [19]. However, unlike in the model used by Weissman *et al.* [19], these growth defects are not caused by the costs of construction and maintenance of the CRISPR-Cas system, but rather originate from the system’s efficiency. Indeed, consistent with the inability of the CRISPR-Cas system to fully block phage gene expression [18], we observed that while the CRISPR-Cas system is efficient enough to prevent bacterial lysis, it cannot prevent other adverse effects, such as SOS response activation and division lag. Therefore, we propose here that the CRISPR immunity inducible costs come from its reduced efficiency compared with SM-resistance at preventing phage-induced stress response and division lag.

## Data Availability

The datasets supporting this article have been uploaded as part of the electronic supplementary material, available online [80] (electronic supplementary material, data S1).

## References

[1] Chevallereau A, Pons BJ, van Houte S, Westra ER. 2022 Interactions between bacterial and phage communities in natural environments. Nat. Rev. Microbiol. **20**, 49–62. (10.1038/s41579-021-00602-y) 34373631

[2] Suttle CA. 2007 Marine viruses — major players in the global ecosystem. Nat. Rev. Microbiol. **5**, 801–812. (10.1038/nrmicro1750) 17853907

[3] Hendrix RW, Smith MCM, Burns RN, Ford ME, Hatfull GF. 1999 Evolutionary relationships among diverse bacteriophages and prophages: all the world’s a phage. Proc. Natl Acad. Sci. USA **96**, 2192–2197. (10.1073/pnas.96.5.2192) 10051617 PMC26759

[4] Hampton HG, Watson BNJ, Fineran PC. 2020 The arms race between bacteria and their phage foes. Nature **577**, 327–336. (10.1038/s41586-019-1894-8) 31942051

[5] Tesson F, Hervé A, Mordret E, Touchon M, d’Humières C, Cury J, Bernheim A. 2022 Systematic and quantitative view of the antiviral arsenal of prokaryotes. Nat. Commun. **13**, 2561. (10.1038/s41467-022-30269-9) 35538097 PMC9090908

[6] Tal N *et al*. 2022 Bacteria deplete deoxynucleotides to defend against bacteriophage infection. Nat. Microbiol. **7**, 1200–1209. (10.1038/s41564-022-01158-0) 35817891

[7] Agapov A *et al*. 2024 Multi-layered genome defences in bacteria. Curr. Opin. Microbiol. **78**, 102436. (10.1016/j.mib.2024.102436) 38368839

[8] Barrangou R, Horvath P. 2017 A decade of discovery: CRISPR functions and applications. Nat. Microbiol. **2**, 17092. (10.1038/nmicrobiol.2017.92) 28581505

[9] Cady KC, Bondy-Denomy J, Heussler GE, Davidson AR, O’Toole GA. 2012 The CRISPR/Cas adaptive immune system of Pseudomonas aeruginosa mediates resistance to naturally occurring and engineered phages. J. Bacteriol. **194**, 5728–5738. (10.1128/jb.01184-12) 22885297 PMC3486085

[10] Marraffini LA. 2015 CRISPR-Cas immunity in prokaryotes. Nature **526**, 55–61. (10.1038/nature15386) 26432244

[11] Westra ER *et al*. 2015 Parasite exposure drives selective evolution of constitutive versus inducible defense. Curr. Biol. **25**, 1043–1049. (10.1016/j.cub.2015.01.065) 25772450

[12] van Houte S *et al*. 2016 The diversity-generating benefits of a prokaryotic adaptive immune system. Nature **532**, 385–388. (10.1038/nature17436) 27074511 PMC4935084

[13] Lenski RE. 1988 Experimental studies of pleiotropy and epistasis in Escherichia coli. I. Variation in competitive fitness among mutants resistant to virus T4. Evolution **42**, 425–432. (10.1111/j.1558-5646.1988.tb04149.x) 28564005

[14] Harvey H, Bondy-Denomy J, Marquis H, Sztanko KM, Davidson AR, Burrows LL. 2017 Pseudomonas aeruginosa defends against phages through type IV pilus glycosylation. Nat. Microbiol. **3**, 47–52. (10.1038/s41564-017-0061-y) 29133883

[15] Buckling A, Brockhurst M. 2012 Bacteria–virus coevolution. In Evolutionary systems biology (ed. O Soyer), pp. 347–370. New York, NY: Springer. (10.1007/978-1-4614-3567-9_16) 22821466

[16] Brockhurst MA, Buckling A, Rainey PB. 2005 The effect of a bacteriophage on diversification of the opportunistic bacterial pathogen, Pseudomonas aeruginosa. Proc. R. Soc. B **272**, 1385–1391. (10.1098/rspb.2005.3086) PMC1560335 16006335

[17] Vale PF, Lafforgue G, Gatchitch F, Gardan R, Moineau S, Gandon S. 2015 Costs of CRISPR-Cas-mediated resistance in Streptococcus thermophilus. Proc. R. Soc. B **282**, 20151270. (10.1098/rspb.2015.1270) PMC4528535 26224708

[18] Meaden S, Capria L, Alseth E, Gandon S, Biswas A, Lenzi L, van Houte S, Westra ER. 2021 Phage gene expression and host responses lead to infection-dependent costs of CRISPR immunity. ISME J. **15**, 534–544. (10.1038/s41396-020-00794-w) 33011743 PMC8027618

[19] Weissman J, Alseth EO, Meaden S, Westra ER, Fuhrman JA. 2021 Immune lag is a major cost of prokaryotic adaptive immunity during viral outbreaks. Proc. R. Soc. B **288**, 20211555. (10.1098/rspb.2021.1555) PMC8527200 34666523

[20] Watson BNJ, Pursey E, Gandon S, Westra ER. 2023 Transient eco-evolutionary dynamics early in a phage epidemic have strong and lasting impact on the long-term evolution of bacterial defences. PLoS Biol. **21**, e3002122. (10.1371/journal.pbio.3002122) 37713428 PMC10530023

[21] Attrill EL, Claydon R, Łapińska U, Recker M, Meaden S, Brown AT, Westra ER, Harding SV, Pagliara S. 2021 Individual bacteria in structured environments rely on phenotypic resistance to phage. PLoS Biol. **19**, e3001406. (10.1371/journal.pbio.3001406) 34637438 PMC8509860

[22] Attrill EL, Łapińska U, Westra ER, Harding SV, Pagliara S. 2023 Slow growing bacteria survive bacteriophage in isolation. ISME Commun. **3**, 95. (10.1038/s43705-023-00299-5) 37684358 PMC10491631

[23] Ugolini GS, Wang M, Secchi E, Pioli R, Ackermann M, Stocker R. 2024 Microfluidic approaches in microbial ecology. Lab Chip **24**, 1394–1418. (10.1039/d3lc00784g) 38344937 PMC10898419

[24] Allard P, Papazotos F, Potvin-Trottier L. 2022 Microfluidics for long-term single-cell time-lapse microscopy: advances and applications. Front. Bioeng. Biotechnol. **10**, 968342. (10.3389/fbioe.2022.968342) 36312536 PMC9597311

[25] O’Toole GA, Kolter R. 1998 Flagellar and twitching motility are necessary for Pseudomonas aeruginosa biofilm development. Mol. Microbiol. **30**, 295–304. (10.1046/j.1365-2958.1998.01062.x) 9791175

[26] Hmelo LR *et al*. 2015 Precision-engineering the Pseudomonas aeruginosa genome with two-step allelic exchange. Nat. Protoc. **10**, 1820–1841. (10.1038/nprot.2015.115) 26492139 PMC4862005

[27] Budzik JM, Rosche WA, Rietsch A, O’Toole GA. 2004 Isolation and characterization of a generalized transducing phage for Pseudomonas aeruginosa strains PAO1 and PA14. J. Bacteriol. **186**, 3270–3273. (10.1128/jb.186.10.3270-3273.2004) 15126493 PMC400619

[28] Tammam S, Sampaleanu LM, Koo J, Manoharan K, Daubaras M, Burrows LL, Howell PL. 2013 PilMNOPQ from the Pseudomonas aeruginosa type IV pilus system form a transenvelope protein interaction network that interacts with PilA. J. Bacteriol. **195**, 2126–2135. (10.1128/jb.00032-13) 23457250 PMC3650547

[29] Kropinski AM. 2009 Measurement of the rate of attachment of bacteriophage to cells. In Bacteriophages (eds MR Clokie, AM Kropinski), pp. 151–155. Totowa, NJ: Humana Press. (10.1007/978-1-60327-164-6_15) 19066819

[30] Choi KH, Schweizer HP. 2006 mini-Tn7 insertion in bacteria with single attTn7 sites: example Pseudomonas aeruginosa. Nat. Protoc. **1**, 153–161. (10.1038/nprot.2006.24) 17406227

[31] Pagliara S, Persano L, Camposeo A, Cingolani R, Pisignano D. 2007 Registration accuracy in multilevel soft lithography. Nanotechnology **18**, 175302. (10.1088/0957-4484/18/17/175302)

[32] Łapińska U *et al*. 2022 Fast bacterial growth reduces antibiotic accumulation and efficacy. eLife **11**, e74062. (10.7554/elife.74062) 35670099 PMC9173744

[33] Pagliara S, Chimerel C, Langford R, Aarts DGAL, Keyser UF. 2011 Parallel sub-micrometre channels with different dimensions for laser scattering detection. Lab Chip **11**, 3365. (10.1039/c1lc20399a) 21804971

[34] Łapińska U *et al*. 2023 Systematic comparison of unilamellar vesicles reveals that archaeal core lipid membranes are more permeable than bacterial membranes. PLoS Biol. **21**, e3002048. (10.1371/journal.pbio.3002048) 37014915 PMC10072491

[35] Cama J, Voliotis M, Metz J, Smith A, Iannucci J, Keyser UF, Tsaneva-Atanasova K, Pagliara S. 2020 Single-cell microfluidics facilitates the rapid quantification of antibiotic accumulation in Gram-negative bacteria. Lab Chip **20**, 2765–2775. (10.1039/d0lc00242a) 32613221 PMC7953842

[36] Stone MRL, Łapińska U, Pagliara S, Masi M, Blanchfield JT, Cooper MA, Blaskovich MAT. 2020 Fluorescent macrolide probes – synthesis and use in evaluation of bacterial resistance. RSC Chem. Biol. **1**, 395–404. (10.1039/d0cb00118j) 34458770 PMC8341779

[37] Zhang Y, Kepiro I, Ryadnov MG, Pagliara S. 2023 Single cell killing kinetics differentiate phenotypic bacterial responses to different antibacterial classes. Microbiol. Spectr. **11**, e0366722. (10.1128/spectrum.03667-22) 36651776 PMC9927147

[38] Glover G *et al*. 2022 Nutrient and salt depletion synergistically boosts glucose metabolism in individual Escherichia coli cells. Commun. Biol. **5**, 385. (10.1038/s42003-022-03336-6) 35444215 PMC9021252

[39] Łapińska U, Glover G, Capilla-Lasheras P, Young AJ, Pagliara S. 2019 Bacterial ageing in the absence of external stressors. Phil. Trans. R. Soc. B **374**, 20180042. (10.1098/rstb.2018.0442) PMC6792439 31587633

[40] Dimitriu T, Kurilovich E, Łapińska U, Severinov K, Pagliara S, Szczelkun MD, Westra ER. 2022 Bacteriostatic antibiotics promote CRISPR-Cas adaptive immunity by enabling increased spacer acquisition. Cell Host Microbe **30**, 31–40. (10.1016/j.chom.2021.11.014) 34932986

[41] Kraus S *et al*. 2024 Phage-induced efflux down-regulation boosts antibiotic efficacy. PLoS Pathog. **20**, e1012361. (10.1371/journal.ppat.1012361) 38941361 PMC11239113

[42] Jones TH, Vail KM, McMullen LM. 2013 Filament formation by foodborne bacteria under sublethal stress. Int. J. Food Microbiol. **165**, 97–110. (10.1016/j.ijfoodmicro.2013.05.001) 23727653

[43] Rizzo MG, De Plano LM, Franco D. 2020 Regulation of filamentation by bacteria and its impact on the productivity of compounds in biotechnological processes. Appl. Microbiol. Biotechnol. **104**, 4631–4642. (10.1007/s00253-020-10590-3) 32246162

[44] Karasz DC, Weaver AI, Buckley DH, Wilhelm RC. 2022 Conditional filamentation as an adaptive trait of bacteria and its ecological significance in soils. Environ. Microbiol. **24**, 1–17. (10.1111/1462-2920.16228) 34929753

[45] Campey A, Łapińska U, Chait R, Tsaneva-Atanasova K, Pagliara S. 2024 Antibiotic resistant bacteria survive treatment by doubling while shrinking. mBio **15**, e0237524. (10.1128/mbio.02375-24) 39565111 PMC11633386

[46] D’Ari R, Huisman O. 1983 Novel mechanism of cell division inhibition associated with the SOS response in Escherichia coli. J. Bacteriol. **156**, 243–250. (10.1128/jb.156.1.243-250.1983) 6352679 PMC215076

[47] Baharoglu Z, Mazel D. 2014 SOS, the formidable strategy of bacteria against aggressions. FEMS Microbiol. Rev. **38**, 1126–1145. (10.1111/1574-6976.12077) 24923554

[48] Torres-Barceló C, Kojadinovic M, Moxon R, MacLean RC. 2015 The SOS response increases bacterial fitness, but not evolvability, under a sublethal dose of antibiotic. Proc. R. Soc. B **282**, 20150885. (10.1098/rspb.2015.0885) PMC4614765 26446807

[49] Sergueev K, Yu D, Austin S, Court D. 2001 Cell toxicity caused by products of the pL operon of bacteriophage lambda. Gene **272**, 227–235. (10.1016/s0378-1119(01)00535-2) 11470529

[50] Simmons EL, Bond MC, Koskella B, Drescher K, Bucci V, Nadell CD. 2020 Biofilm structure promotes coexistence of phage-resistant and phage-susceptible bacteria. mSystems **5**, e00877-19. (10.1128/mSystems.00877-19) 32576653 PMC7311319

[51] Burmølle M, Ren D, Bjarnsholt T, Sørensen SJ. 2014 Interactions in multispecies biofilms: do they actually matter? Trends Microbiol. **22**, 84–91. (10.1016/j.tim.2013.12.004) 24440178

[52] Berryhill BA, Garcia R, McCall IC, Manuel JA, Chaudhry W, Petit MA, Levin BR. 2023 The book of Lambda does not tell us that naturally occurring lysogens of Escherichia coli are likely to be resistant as well as immune. Proc. Natl Acad. Sci. USA **120**, e2212121120. (10.1073/pnas.2212121120) 36881631 PMC10089163

[53] Murata T. 2014 Regulation of Epstein-Barr virus reactivation from latency. Microbiol. Immunol. **58**, 307–317. (10.1111/1348-0421.12155) 24786491

[54] Bulssico J, PapukashvilI I, Espinosa L, Gandon S, Ansaldi M. 2023 Phage-antibiotic synergy: cell filamentation is a key driver of successful phage predation. PLoS Pathog. **19**, e1011602. (10.1371/journal.ppat.1011602) 37703280 PMC10519598

[55] Foster PL. 2007 Stress-induced mutagenesis in bacteria. Crit. Rev. Biochem. Mol. Biol. **42**, 373–397. (10.1080/10409230701648494) 17917873 PMC2747772

[56] Andersson DI, Hughes D. 2014 Microbiological effects of sublethal levels of antibiotics. Nat. Rev. Microbiol. **12**, 465–478. (10.1038/nrmicro3270) 24861036

[57] Campoy S, Hervàs A, Busquets N, Erill I, Teixidó L, Barbé J. 2006 Induction of the SOS response by bacteriophage lytic development in Salmonella enterica. Virology **351**, 360–367. (10.1016/j.virol.2006.04.001) 16713610

[58] Willner DL, Paudel S, Halleran AD, Solini GE, Gray V, Saha MS. 2024 Transcriptional dynamics during Rhodococcus erythropolis infection with phage WC1. BMC Microbiol. **24**, 107. (10.1186/s12866-024-03241-4) 38561651 PMC10986025

[59] Colasanti J, Denhardt DT. 1985 Expression of the cloned bacteriophage phi X174 A* gene in Escherichia coli inhibits DNA replication and cell division. J. Virol. **53**, 807–813. (10.1128/jvi.53.3.807-813.1985) 3156255 PMC254711

[60] Greer H. 1975 The kil gene of bacteriophage lambda. Virology **66**, 589–604. (10.1016/0042-6822(75)90231-7) 1098278

[61] Haeusser DP, Hoashi M, Weaver A, Brown N, Pan J, Sawitzke JA, Thomason LC, Court DL, Margolin W. 2014 The Kil peptide of bacteriophage λ blocks Escherichia coli cytokinesis via ZipA-dependent inhibition of FtsZ assembly. PLoS Genet. **10**, e1004217. (10.1371/journal.pgen.1004217) 24651041 PMC3961180

[62] Boeckh C, Bade EG, Delius H, Reeve JN. 1986 Inhibition of bacterial segregation by early functions of phage mu and association of replication protein B with the inner cell membrane. Mol. Gen. Genet. **202**, 461–466. (10.1007/BF00333277) 3520239

[63] Conter A, Bouché JP, Dassain M. 1996 Identification of a new inhibitor of essential division gene ftsZ as the kil gene of defective prophage Rac. J. Bacteriol. **178**, 5100–5104. (10.1128/jb.178.17.5100-5104.1996) 8752325 PMC178304

[64] de Boer PA. 2010 Advances in understanding E. coli cell fission. Curr. Opin. Microbiol. **13**, 730–737. (10.1016/j.mib.2010.09.015) 20943430 PMC2994968

[65] Erickson HP, Anderson DE, Osawa M. 2010 FtsZ in bacterial cytokinesis: cytoskeleton and force generator all in one. Microbiol. Mol. Biol. Rev. **74**, 504–528. (10.1128/mmbr.00021-10) 21119015 PMC3008173

[66] Malone LM, Hampton HG, Morgan XC, Fineran PC. 2022 Type I CRISPR-Cas provides robust immunity but incomplete attenuation of phage-induced cellular stress. Nucleic Acids Res. **50**, 160–174. (10.1093/nar/gkab1210) 34928385 PMC8754663

[67] Heussler GE, Cady KC, Koeppen K, Bhuju S, Stanton BA, O’Toole GA. 2015 Clustered regularly interspaced short palindromic repeat-dependent, biofilm-specific death of Pseudomonas aeruginosa mediated by increased expression of phage-related genes. mBio **6**, e00129-15. (10.1128/mBio.00129-15) 25968642 PMC4436051

[68] Dharmalingam K, Goldberg EB. 1980 Restriction in vivo. V. Introduction of SOS functions in Escherichia coli by restricted T4 phage DNA, and alleviation of restriction by SOS functions. Mol. Gen. Genet. **178**, 51–58. (10.1007/BF00267212) 6991879

[69] Hayes S, Erker C, Horbay MA, Marciniuk K, Wang W, Hayes C. 2013 Phage lambda P protein: trans-activation, inhibition phenotypes and their suppression. Viruses **5**, 619–653. (10.3390/v5020619) 23389467 PMC3640518

[70] de Boer PA, Crossley RE, Rothfield LI. 1990 Central role for the Escherichia coli minC gene product in two different cell division-inhibition systems. Proc. Natl Acad. Sci. USA **87**, 1129–1133. (10.1073/pnas.87.3.1129) 2137246 PMC53424

[71] Ragunathan PT, Vanderpool CK. 2019 Cryptic-prophage-encoded small protein DicB protects Escherichia coli from phage infection by inhibiting inner membrane receptor proteins. J. Bacteriol. **201**, e00475-19. (10.1128/JB.00475-19) 31527115 PMC6832061

[72] Ragunathan PT, Ng Kwan Lim E, Ma X, Massé E, Vanderpool CK. 2023 Mechanisms of regulation of cryptic prophage-encoded gene products in Escherichia coli. J. Bacteriol. **205**, e0012923. (10.1128/jb.00129-23) 37439671 PMC10448788

[73] Stewart CR, Deery WJ, Egan ESK, Myles B, Petti AA. 2013 The product of SPO1 gene 56 inhibits host cell division during infection of Bacillus subtilis by bacteriophage SPO1. Virology **447**, 249–253. (10.1016/j.virol.2013.09.005) 24210121

[74] Zhang P *et al*. 2022 Bacteriophage protein Gp46 is a cross-species inhibitor of nucleoid-associated HU proteins. Proc. Natl Acad. Sci. USA **119**, e2116278119. (10.1073/pnas.2116278119) 35193978 PMC8892312

[75] Tollrian R, Harvell CD. 1999 The evolution of inducible defenses: current ideas. In The ecology and evolution of inducible defenses. 1st edn (eds R Tollrian, CD Harvell), pp. 306–322. Princeton, NJ: Princeton University Press. (10.2307/j.ctv1ddd1cn.22)

[76] Kraaijeveld AR, Godfray HCJ. 1997 Trade-off between parasitoid resistance and larval competitive ability in Drosophila melanogaster. Nature **389**, 278–280. (10.1038/38483) 9305840

[77] Boots M, Begon M. 1993 Trade-offs with resistance to a granulosis virus in the Indian meal moth, examined by a laboratory evolution experiment. Funct. Ecol. **7**, 528–534. (10.2307/2390128)

[78] Moret Y, Schmid-Hempel P. 2000 Survival for immunity: the price of immune system activation for bumblebee workers. Science **290**, 1166–1168. (10.1126/science.290.5494.1166) 11073456

[79] Harvell CD. 1990 The ecology and evolution of inducible defenses. Q. Rev. Biol. **65**, 323–340. (10.1086/416841) 2236483

[80] Pons BJ, Lapinska U, Lopes-Domingues I, Chisnall MAW, Westra E, Pagliara S *et al*. 2025 Supplementary material from: Phage provoke growth delays and SOS response induction despite CRISPR-Cas protection. Figshare (10.6084/m9.figshare.c.7947691) PMC12409350 40904102

